# On taylor correlation functions in isotropic turbulent flows

**DOI:** 10.1038/s41598-023-30825-3

**Published:** 2023-03-08

**Authors:** Wei Chen

**Affiliations:** 1 Department of Applied Mathematics, University of Science and Technology Beijing, 30 Xueyuan Rd, Beijing, China; 2 General Electric, 300 Garlington Rd, Greenville, South Carolina USA

**Keywords:** Applied physics, Aerospace engineering

## Abstract

A turbulent flow can be characterized by Taylor correlation functions which are obtained empirically, understood by statistical mechanics and regarded as universal. Here, we show that Taylor correlations are analytically derived by hypothesizing turbulence as a phenomenon of superfluids at resonance. Leveraging from a recent study on heat transfer at the speed of sound, we derived and fitted the longitudinal and lateral turbulent velocities in an isotropic, turbulent flow. The concept of the boundary of the second law helps to specify the integration constants in the solution. From the velocity profiles, Taylor’s correlation functions are analytically determined. From the linearity of the eigenfunction, we introduce amplitude and frequency factors. These factors are curve-fitted with two experimental dataset. Additional experimental datasets in the public domain are compared to the correlations, which shows that the theory agrees with experiments very well in isotropic flows. The analytical correlation functions help to elucidate observations that experiments and statistical mechanics have challenges to explain.

## Introduction

In dealing with practical, turbulent flow problems, empirical correlations and coefficients are inevitable regardless of whether computational or analytical methods are used^1–5^. Taylor correlation functions are probably the first used to characterize the structures in turbulent flows and are considered to be universal^2,4,6–8^. In this work, by hypothesizing that a turbulent flow is a generic problem of superfluid at resonance, we analytically derive and validate these correlations with experimental data.

Fundamentally, turbulence is an interdisciplinary topic since it has been observed in fluid flows^4^, electromagnetic fields^9^ and ballistic heat transfer^10^. Turbulence in these different subjects must share a common fundamental physics. Revelation of such physics should provide a key to the solutions of all turbulence problems. In a turbulent flow, one of the most striking attributes is elevated sound generation^11,12^. A recent study on heat transfer at the speed of sound established the connection between superfluid and sound by thermodynamics, not only at low temperatures^13^. In a fluid domain, superfluid is dynamically and isentropically formed locally and temporarily. Moreover, the resonant superfluid generates the first and the second sound. Hence, sound is the proxy of resonant superfluid universally. The study hypothesizes that a viscous fluid locally turns into a superfluid where the excess entropy reaches zero in a periodic motion. The excess entropy, $\delta {s}=s_{ND}-s_D$, is defined as the subtraction of the mass density of entropy of a dissipative fluid, $s_{D}$, from that of a non-dissipative fluid, $s_{ND}$. Here, the terms of viscous, dissipative and regular fluids are interchangeable, as are superfluid and non-dissipative fluids.

An important finding in that study is that when excess entropy becomes zero at any given time or location, there is a change in the thermodynamic state from entropic to isentropic. Correspondingly, isentropic flows follow the Euler equations instead of the Navier-Stokes equations. Since the occurrences of the isentropic state in a viscous fluid are due to the constraint of the second law, those special time periods and spatial domains are called on the boundary of the second law by^13^. The physics revealed by the excess entropy is far beyond the fluid dynamics and implies that any dissipative dynamics has corresponding non-dissipative dynamics. While we continue to make progress in understanding the scope of this physics, it becomes clear that the entire family of the consistent fundamental physics on isentropic motion has been overlooked. Such an attainment indicates that the quasi steady state solutions at interfaces^13^ and the spontaneity of helium flows^14^ share a class of solutions across disciplines. Similarly, the work in this manuscript on the transient velocities of resonant superfluid establishes the method on how to define boundary conditions and solve the transient resonant super state in physics, chemistry, biology, electromagnetism and geology etc.

The boundary of the second law has the same function as any physical boundary. It separates superfluid from a regular fluid, has different governing equations on both sides and allows the exchange of mass, momentum and energy to maintain conservation. On the other hand, it is unique since its existence depends on the motion and happens on whether the constraint of the second law is reached. To emphasize these properties, it is called the boundary of the second law in a flow field where the excess entropy reaches zero.

Local and repetitive occurrences of superfluid lead to the resonance of the entire flow system. With the elimination of nonlinear terms, the study derived the governing equations of resonant superfluids^13^, where the resonance was determined by the Jacobian matrix, a technique from Landau^15^ given in Supplement:

1 $$\begin{aligned}{} & {} \frac{D\rho }{Dt}+\rho {{\mathbf {\nabla }}}\cdot {\textbf{u}}=0 \end{aligned}$$

2 $$\begin{aligned}{} & {} \quad \rho \left( \frac{\partial \textbf{u}}{\partial {t}}\pm {{\mathbf {c}}_1}\cdot \nabla {\textbf{u}}\right) = -\nabla {p} \end{aligned}$$

3 $$\begin{aligned}{} & {} \quad \rho \left( \frac{\partial {T}}{\partial {t}}\pm {{\mathbf {c}}_2}\cdot \nabla {T}\right) = 0 \end{aligned}$$

where $\rho$,$\textbf{u}$, *T*, *p*, ${{\mathbf {c}}_1}=c_1\textbf{i}+c_1\textbf{j}$ and ${{\mathbf {c}}_2}=c_2\textbf{i}+c_2\textbf{j}$ are the density, velocity vector, temperature, pressure, the first and second sound vectors and $\textbf{i}$ and $\textbf{j}$ are the unit vectors. The plus and minus sign indicates that sound can propagate in both directions.

In this manuscript, we first solve the differential equations of the momentum conservation (Eq. 2) for superfluid at resonance. Owing to the constant speed of sound, the derivative of the velocity of the superfluid depends linearly on time and space variables through the eigenfunction of Eq. (2). This linear dependence greatly simplifies the mathematics in searching for solutions and permits the casting of velocities in time only. With the assumption of isotropic turbulence, the boundary conditions are used to specify the constants in the general solution of velocities on the boundary of the second law. Since the concept of the boundary of the second law is new, we go into fair detail to demonstrate how to apply them. In the determination of boundary conditions, we introduce the Dirac comb to quantitatively represent impulses at the transition of thermodynamic states. The solution of superfluid at resonance consists of two parts: transient and quasi steady-state. At resonance, the quasi steady state motion immediately participates in resonance and loses its identity of impulses. Hence, we focus on the transient velocity fields, which are the turbulent velocities. The longitudinal and lateral correlations are derived from the turbulent velocity fields. Since it does not alter the characteristics of velocities due to the linearity of eigenfunctions, we introduce the amplitude factors and frequency factors to recover the experimental data. We quantify the coefficients of the correlations from the experimental data available. Then, we compare these correlations to the experimental data from other sources. In particular, we compare the longitudinal correlation to a dataset with spatial measurements. In that comparison, we use the same factors to calculate the correlation on the negative spatial parameter. The theory produces good agreement in spatial and time delays. Additional benefits of curve fitting are the availability of velocity distributions, and we discuss velocity distributions and explain some phenomena that statistical mechanics falls short. The conclusion summarizes the key findings and our perspective for future work.

## Equations of motion

The goal of this section is to derive a general velocity field in an isotropic turbulent flow. An isotropic turbulent flow field such as these behind grid flow can be treated as a two-dimensional flow, where the longitudinal velocity in the x-axis is in the main flow direction and the lateral velocity in the y-axis represents other two directions. The differential equations are readily available from Eq. (2) for the positive characteristic velocity, $c_1$,

4 $$\begin{aligned}{} & {} \frac{\partial u}{\partial t}+c_1\left( \frac{\partial u}{\partial x}+\frac{\partial u}{\partial y}\right) = -\frac{1}{\rho } \frac{\partial p}{\partial x} \end{aligned}$$

5 $$\begin{aligned}{} & {} \quad \frac{\partial v}{\partial t}+c_1\left( \frac{\partial v}{\partial x}+\frac{\partial v}{\partial y}\right) = -\frac{1}{\rho } \frac{\partial p}{\partial y} \end{aligned}$$

where *u* and *v* are the longitudinal and lateral turbulent velocities, respectively. *p* and $\rho$ are the pressure and density, respectively. Since it does not depend on the lateral velocity, we can focus on the longitudinal velocity first. A superfluid at resonance has two velocity components, transient and quasi-steady state and we are more interested in the solution of the transient part. By assuming constant speed of sound, the first order,linear partial different equations can be represented by the superposition of homogeneous and inhomogeneous parts, $u=u_h+u_p$^16^. The homogeneous differential equation of Eq. (4) is given

6 $$\begin{aligned} \frac{\partial u_h}{\partial t}+c_1\left( \frac{\partial u_h}{\partial x}+\frac{\partial u_h}{\partial y}\right) = 0 \end{aligned}$$

Denote $u_h=A(x)B(y)C(t)$ for the separation method to Eq. (6),

7 $$\begin{aligned} AB\frac{\partial C}{\partial t}+{c_1}BC\frac{\partial A}{\partial x}+{c_1}AC\frac{\partial B}{\partial y} = 0 \end{aligned}$$

Divide Eq. (7) by $u_h$,

8 $$\begin{aligned} {\frac{1}{C}}\frac{dC}{dt}+{\frac{1}{A}}\frac{dA}{dx}+{\frac{1}{B}}\frac{dB}{dy} = 0 \end{aligned}$$

Since each term in Eq. (8) is the sole function of *x*, *y*, *t*, Eq. (8) can be written as

9 $$\begin{aligned} -n_1+n_2+n_3 = 0 \end{aligned}$$

where $n_i, i=1,2,3$ are complex eigenvalues and are constants for time, *x*, and *y*, respectively. The eigenfunction (Eq. 9) has many useful and important properties. In this work, we benefit from three of them. First, the linear dependence of velocity on time and space variables simplifies the velocity fields and allows us to solve velocity fields on time only. Second, Eq. (9) can be multiplied by any nonzero constant without changing the nature of velocity fields, which helps to curve-fit correlations. Third, the complex eigenvalues yield two independent, equivalent eigenfunctions for real and imaginary parts. With this property, we can treat the transient part of the solution as the turbulent field, where the eigenvalues for real parts are not zero. The quasi steady state part sustains the resonance since its eigenvalue for the real part is zero and does not decay.

The separation method yields the general homogeneous solution,

10 $$\begin{aligned} u_h = \sum \limits _{n_1=1}^NC_{0,n_1}\sum \limits _{n_2=0}^{\pm N}A_{0,n_2}\sum \limits _{n_3=0}^{\pm N}B_{0,n_3}e^{-n_1t}e^{\frac{\pm n_2x}{c_1}}e^{\frac{\pm n_3y}{c_1}} \end{aligned}$$

where we use the shorthand *N* and its summation to replace the double summation on the real and imaginary parts separately and $A_{0,n_2}$, $B_{0,n_3}$ and $C_{0,n_1}$ are integration constants to be determined by boundary conditions. Since the flow is dominated in *x* direction, the pressure is assumed a linear function longitudinally $p=e^{-n_1 t}(\alpha _0 x+\beta _0)$ for convenience. We obtain the inhomogeneous solutions,

11 $$\begin{aligned} u_p = \sum \limits _{n_1=1}^Ne^{-n_1t}\left( -b_0\frac{c_1}{n_1}e^{\frac{ n_1x}{c_1}}+\frac{\alpha _0}{n_1 \rho } \right) \end{aligned}$$

where $\alpha _0$ is a constant determined by the pressure drop of the given viscous flow and $b_0$ is the coefficient of the series to be determined by the boundary conditions. The solution of the transient part is arrived at

12 $$\begin{aligned} \begin{aligned} u =&\sum \limits _{n_1=1}^NC_{0,n_1}e^{-n_1t}\left[ \sum \limits _{n_2=0}^{\pm N}A_{0,n_2} \right. \\&\left. \sum \limits _{n_3=0}^{\pm N}B_{0,n_3}e^{\frac{\pm n_2x}{c_1}}e^{\frac{\pm n_3y}{c_1}}+ \phi _{n_1}\right] \end{aligned} \end{aligned}$$

where $\phi _{n_1}=\frac{1}{C_{0,n_1}}\left( -b_0\frac{c_1}{n_1}e^{\frac{ n_1x}{c_1}}+\frac{\alpha _0}{n_1 \rho } \right)$. If one only focus on the temporal component, the real part of Eq. (12) becomes

13 $$\begin{aligned} u = \sum \limits _{k=1}^K\left( {T_{1,k}\cos { kt}+T_{2,k}\sin {kt}}\right) e^{-kt} \end{aligned}$$

where $T_{1,k}$ and $T_{2,k}$ are the real coefficients to be determined by boundary conditions and *k* and *K* are real as well. Similarly, we can obtain the lateral velocity

14 $$\begin{aligned} v = \sum \limits _{l=1}^L\left( {U_{1,l}\cos { l t}+U_{2,l}\sin {lt}}\right) e^{-lt} \end{aligned}$$

where $U_{1,l}$ and $U_{2,l}$ are the real coefficients to be determined by boundary conditions and *l* and *L* are real as well, which are generally not equal to *k* and *K*. Here, it should be noted that the quasi steady state solution has the eigenvalues of $n_1=k+0i$, which yields the solution in^13^ of

15 $$\begin{aligned} -p_x =\pm \rho {c_1 u_s} \quad \textrm{and}\quad -p_y =\pm \rho {c_1 v_s} \end{aligned}$$

where $c_1$ is the speed of the first sound and $u_s$ and $v_s$ are steady state velocities and can be expressed by sinusoidal waves,

16 $$\begin{aligned} u_s= {} \sum \limits _{k=1}^K\left( {T_{3,k}\cos { kt}+T_{4,k}\sin {kt}}\right) \end{aligned}$$

17 $$\begin{aligned} v_s= {} \sum \limits _{l=1}^L\left( {U_{3,l}\cos { l t}+U_{4,l}\sin {lt}}\right) \end{aligned}$$

where $T_{3,k}$, $T_{4,k}$ and $U_{3,l}$ and $U_{4,l}$ are the coefficients to be determined by resonance and steady state boundary conditions. Here, we want to clarify the difference between superfluid at resonance and turbulence, where superfluid at resonance includes both transient and steady state velocity fields but a turbulent flow is only the transient portion. At its generation, the quasi steady state portion loses its identity from Eq. (4) and becomes the part of resonance. Since we only consider turbulence, we will only focus on the transient portion of the solution in this manuscript. We obtain the turbulent velocity Eqs. (13) and (14) and may employ frequency instead of angular frequency for convenience,

18 $$\begin{aligned} u= {} \sum \limits _{k=1}^K\left( {T_{1,k}\cos {2\pi {kt}}+T_{2,k}\sin {2\pi {kt}}}\right) e^{-2\pi {kt}} \end{aligned}$$

19 $$\begin{aligned} v= {} \sum \limits _{l=1}^L\left( {U_{1,l}\cos {2\pi {l t}}+U_{2,l}\sin {2\pi {lt}}}\right) e^{-2\pi {lt}} \end{aligned}$$

In summary, we obtained the turbulent velocities in an isotropic, multi-dimensional field. Although a turbulent field is three-dimensional, we can express them in time only because of the isotropic nature of the field. Both longitudinal and lateral solutions of superfluid at resonance have two parts. A transient part represents the response to the impulses and the decay with time, and a quasi-steady state part sustains the resonance. A turbulent flow field takes only the transient part of the superfluid at resonance. To derive the Taylor correlation functions for turbulence, we will solve these coefficients in the transient part Eqs. (18) and (19) in the next section.

## Boundary of second law

The purpose of this section is to determine the integration constants in the general solutions of turbulent velocities. Since it arises in a regular fluid, a superfluid is surrounded by the regular fluid, and the interface between the regular fluid and superfluid is the boundary. Therefore, the boundary condition must be established between the superfluid and the regular fluid. In this section, we quantitatively establish this new kind of boundary condition through the concept of the boundary of the second law. In the transition of thermodynamic states, the physical process begins when the excess entropy becomes zero, which means that at that very short and ephemeral moment, a fluid parcel experiences isentropic flow. Since an isentropic flow is non-dissipative, this fluid parcel at that particular moment appears to have no viscosity and becomes superfluid, similar to the “fixed” and “movable” spots by^17^. Consequently, we must supply the Euler equations to describe the motion of this fluid parcel, not the Navier-Stokes equations. Since this instant is so short that the representation of this change is best described by an impulse. If these instants occur continuously, we will have a row of impulses, which is mathematically called the Dirac comb. In this section, we use the Dirac comb as a tool to determine the integration constants step by step.

The theory of excess entropy relies on the second law of thermodynamics, which states that a non-dissipative process does not incur any loss due to viscosity. This law holds anywhere and any moment, and there is no exception infinitesimally. Then, we should be able to describe it by the differential equations of fluid motion. Since the excess entropy is the difference of entropy between the dissipative motion, $s_{D}$, to a non-dissipative motion, $s_{ND}$,

20 $$\begin{aligned} \delta {s}=s_{ND}-s_D \end{aligned}$$

If the excess entropy is zero in space or time, the flow becomes non-dissipative at that instant. Hence, at that instant, the flow appears to have no viscosity and no thermal conductivity and follows the Euler equations. Derived from the energy equation, the excess entropy can be calculated by the differential equation below:

21 $$\begin{aligned} \frac{\partial \delta {s}}{\partial t}+\textbf{u}^\dagger \cdot \nabla \delta {s} =\frac{1}{\rho T}{\mathbf {\nabla }}\cdot \textbf{q}-\frac{\varepsilon }{\rho T} \end{aligned}$$

where $\textbf{u}^\dagger$ is the velocity vector in the regular fluid and ${\mathbf {\nabla }}\cdot \textbf{q}$ denotes the divergence of heat flux and $\varepsilon ={\mathbf {\tau }}:{\mathbf {\nabla }}\textbf{u}$, dissipation due to viscosity. ${\mathbf {\tau }}=-\mu \left( {\mathbf {\nabla }}\textbf{u}+({\mathbf {\nabla }}\textbf{u})^T \right) -(\zeta -\frac{2}{3}\mu )({\mathbf {\nabla }}\cdot \textbf{u})I$ is the stress tensor, $\zeta$ is bulk viscosity and $\mu$ is molecular viscosity. If we assume that there is no heat conduction, to guarantee that excess entropy equals zero, the dissipation term must be zero.

22 $$\begin{aligned} \varepsilon = 0 \end{aligned}$$

For an isotropic turbulent flow, we also assume the regular fluid side can be described by a two-dimensional flow and explicitly write (Eq. 22) on the velocity gradient with constant viscosity,

23 $$\begin{aligned} \begin{aligned} \varepsilon&=2\left[ {\left( \frac{\partial u^\dagger }{\partial x}\right) }^2+{\left( \frac{\partial v^\dagger }{\partial x}\right) }^2\right] +{\left[ \frac{\partial v^\dagger }{\partial x}+\frac{\partial u^\dagger }{\partial y}\right] }^2 \\& \quad -\frac{2}{3}{\left[ \frac{\partial u^\dagger }{\partial x}+\frac{\partial v^\dagger }{\partial y}\right] }^2=0 \end{aligned} \end{aligned}$$

If we assume that there is a small perturbation in velocity, they are

24 $$\begin{aligned} \begin{aligned} u^\dagger&= u^\dagger _0 \sum \limits _{n,n_1,n_2=1}^Na_n \sin {2\pi n_1 x} \sin {2\pi n_2 y} \sin {2\pi n t} \\&= \phi (x,y) \alpha (t) \end{aligned} \end{aligned}$$

25 $$\begin{aligned} \begin{aligned} v^\dagger&= v^\dagger _0 \sum \limits _{n,n_1,n_2=1}^Nb_n \sin {2\pi n_1 x} \sin {2\pi n_2 y} \cos {2\pi n t} \\&= \pi (x,y) \beta (t) \end{aligned} \end{aligned}$$

where $n_i, i=1,2,3$ are the harmonics of the perturbations and $u^\dagger _0$ and $v^\dagger _0$ are the amplitudes of the longitudinal and lateral perturbation velocities, respectively. The functions, $\phi (x,y)$ and $\pi (x,y)$ and coefficients $a_n$ and $b_n$ are chosen such that they satisfy the continuity equation. Bring Eqs. (24) and (25) to (23), (22) becomes

26 $$\begin{aligned} \varepsilon =S_1{\alpha ^2}+S_2{\beta ^2}+S_3{\alpha \beta } \end{aligned}$$

where $S_1=2{\left( \frac{\partial \phi }{\partial x} \right) }^2+{\left( \frac{\partial \phi }{\partial y} \right) }^2-\frac{2}{3}{\left( \frac{\partial \phi }{\partial x} \right) }^2$, $S_2={\left( \frac{\partial \pi }{\partial x} \right) }^2+2{\left( \frac{\partial \pi }{\partial y} \right) }^2-\frac{2}{3}{\left( \frac{\partial \pi }{\partial y} \right) }^2$ and $S_3=2(\frac{\partial \pi }{\partial x}\frac{\partial \phi }{\partial y}- \frac{2}{3}\frac{\partial \pi }{\partial y}\frac{\partial \phi }{\partial x})$ are only spatial functions. In an isotropic turbulent field, the boundary of the second law is obtained by

27 $$S_{3} \alpha \beta = S_{3} \sin 2\pi nt\cos 2\pi nt = 0$$

28 $$S_1\alpha ^2= S_1\sin {2\pi {nt}}\sin {2\pi {nt}}=0$$

29 $$S_2\beta ^2= S_2\cos {2\pi {nt}}\cos {2\pi {nt}}=0$$

**Figure 1:**
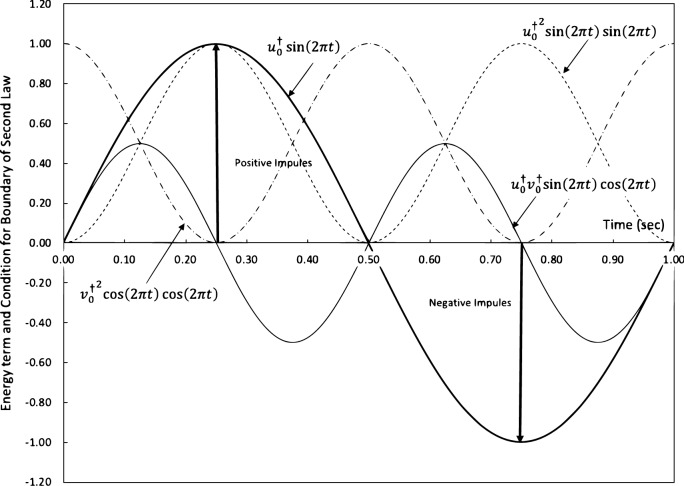
Illustration of the boundary of the second law and impulses in a dirac comb.

Although Eqs. (27)–(29) show three sets of conditions being on the boundary of the second law, there is only one contributing to the boundary conditions. This can be explained by Fig. 1, where we plot 4 curves. The solid heavy curve is the longitudinal perturbation, $u^\dagger = u_0^\dagger \sin {2\pi t}$, and other three curves are on the boundary of the second law. The light solid curve is the condition (Eq. 27) $\alpha \beta =u^\dagger _0 v^\dagger _0\sin {2\pi {t}}\cos {2\pi {t}}$, and both short and long dash curves are Eqs. (28) and (29). For Eq. (27), there are five zero points along time t, and three of them collapse at the same zero locations with the perturbation. Hence, only two of them are effective at approximately $t=0.25$ and 0.75 s. At $t=0.25$ s, a positive impulse is produced and at $t=0.75$ s, a negative impulse is observed and both are represented by heavy lines with arrows. Because at the moment of changing thermodynamic state, the positive impulse is created on the positive peak $u_0^\dagger$ and the negative impulse is produced on the negative peak $-u_0^\dagger$ of $u_0^\dagger \sin {2\pi {t}}$. The other two dash curves have to be zero to guarantee (22), where (29) falls to zero at $t=0.25$ and 0.75 s as shown in Fig. 1 and (28) relies on the spatial term $S_1=0$ at $t=0.25$ and 0.75 s.

As the motion (Eq. 24) continues, the impulses are continuously generated on the boundary of the second law. This array of impulses can be mathematically described by a so-called Dirac comb^18^, which is denoted for the sinusoidal function in Fig. 1 as

30 $$\begin{aligned} \pitchfork _1= u^\dagger _0 \sum \limits _{k=1}^K \delta [t-k+0.75] \end{aligned}$$

31 $$\begin{aligned} \pitchfork _2= u^\dagger _0 \sum \limits _{k=1}^K -\delta [t-k+0.25] \end{aligned}$$

where Eq. (30) is the Dirac comb for the positive impulses and Eq. (31) is for the negative impulses, where the period is assumed 1 and *k* is the period of interest. Equations (30) and (31) represent continuous impulses with time at intervals of period *k*. The Fourier transformation of Eqs. (30) and (31) are readily available for a Dirac comb as

32 $$\begin{aligned} \pitchfork _1 (k)= u^\dagger _0 \sum \limits _{k=1}^K \delta [k] \end{aligned}$$

33 $$\begin{aligned} \pitchfork _2 (k)= u^\dagger _0 \sum \limits _{k=1}^K -\delta [k] \end{aligned}$$

Hence, the coefficients of Fourier transformation of the Dirac combs Eqs. (30) and (31) are

34 $$\begin{aligned} c_1 (k)= u^\dagger _0, \quad k=1,\,\ldots ,\,K \end{aligned}$$

35 $$\begin{aligned} c_2 (k)= -u^\dagger _0, \quad k=1,\,\ldots ,\,K \end{aligned}$$

The independent coefficients of Fourier transformation of Eq. (18) equate to those of Eqs. (34) and (35), which produces

36 $$\begin{aligned} \left\{ \begin{array}{ll} u^\dagger _0 =\int ^\infty _0\left( {T_{1,k}\cos {2\pi kt}+T_{2,k}\sin {2\pi kt}}\right) \\ e^{-2\pi kt}\cos {2\pi kt}{d t} \\ \displaystyle -u^\dagger _0=\int ^\infty _0\left( {T_{1,k}\cos {2\pi kt}+T_{2,k}\sin {2\pi kt}}\right) \\ e^{-2\pi kt}\sin {2\pi kt}{d t} \end{array}\right. \end{aligned}$$

Since $T_{1,k}$ and $T_{2,k}$ are not the function of time, rearrange (Eq. 36)

37 $$\begin{aligned} \left\{ \begin{array}{ll} I_1T_{1,k}+I_2T_{2,k} = u^\dagger _0\\ \displaystyle I_2T_{1,k}+I_3T_{2,k} = -u^\dagger _0 \end{array}\right. \end{aligned}$$

where

$$\begin{aligned} I_1= {} \int ^\infty _0{\cos {2\pi kt}e^{-2\pi kt}\cos {2\pi kt}dt}=\frac{3}{10\pi k}\\ I_2= {} \int ^\infty _0{\sin {2\pi kt}e^{-2\pi kt}\cos {2\pi kt}dt}=\frac{1}{10\pi k}\\ I_3= {} \int ^\infty _0{\sin {2\pi kt}e^{-2\pi kt}\sin {2\pi kt}dt}=\frac{2}{10\pi k} \end{aligned}$$

Solve Eq. (37) and assume $u_0=u^\dagger _0$. We obtain the longitudinal velocity,

38 $$\begin{aligned} u = 2\pi u_0\sum \limits _{k=1}^K{k\left( 3\cos {2\pi {kt}}-4\sin {2\pi {kt}}\right) e^{-2\pi {kt}}} \end{aligned}$$

Similarly, we can derive the lateral turbulent velocity,

39 $$\begin{aligned} v = 2\pi v_0\sum \limits _{l=1}^Ll\left( {3\cos {2\pi {l t}}-4\sin {2\pi {lt}}}\right) e^{-2\pi {lt}} \end{aligned}$$

Now, we have derived the longitudinal and lateral turbulent velocity for an isotropic turbulent flow field closed with the boundary conditions.

Again, the turbulent velocities Eqs. (38) and (39) only represent the transient component of superfluid at resonance since they keep the identities of impulses, which diminish after a short period. On the other hand, the quasi steady state velocities sustain resonance and do not relate to the transient process as soon as they are generated. The turbulent velocities Eqs. (38) and (39) are expressed in series, where each term consists of sine and cosine functions and exponential functions. Although they are only a function of time, the turbulent velocities are also a function of spatial excitation. Such dependence still remains through the relationship of eigenvalues in Eq. (9). During validation, we will show that with small adjustments of the frequency and amplitude factors, the effects of spatial excitation can be included.

In summary, we derive the turbulent velocities in a multidimensional isotropic turbulent flow. When the inevitable, small perturbations, represented by the Fourier series, are used in the differential equation of the excess entropy, we can find the boundaries of the second law. In this section, we showed that it is impossible to keep the perturbations from “touching” the boundary of the second law. These contacts of the thermodynamic boundary create a set of impulses that turn a regular fluid into a superfluid at resonance. From the differential equations (4) and (5), we are able to determine the turbulent velocities Eqs. (38) and (39), which are used to derive the Taylor correlation functions in the next section.

## Correlation functions

In this section, we derive the correlation functions from the velocity distributions Eqs. (38) and (39). The correlation functions will be validated by experimental data in the next section.

Before we proceed in the derivation of the correlation functions, we examine whether the mean and mean square of velocities are consistent with observations. By definition, the mean velocity is calculated by $\overline{u}=\frac{1}{K}\sum \limits _{k=1}^K{\overline{u}_k}$, $\overline{u}_k=\frac{1}{T}\int ^T_0{u_k}dt$, and $u_k={2\pi u_0}{k\left( 3\cos {2\pi kt}-4\sin {2\pi kt}\right) }$ $e^{-2\pi kt}$. From Eq. (38), the following expression for the mean velocity is calculated:

40 $$\begin{aligned} \begin{aligned} \overline{u}&=\lim _{T, K \rightarrow \infty }\frac{1}{TK}\int ^T_0{2\pi u_0}\sum \limits _{k=1}^Kk\left( 3\cos {2\pi kt} \right. \\&\left. -4\sin {2\pi kt}\right) e^{-2\pi kt}\,dt=0 \end{aligned} \end{aligned}$$

From the longitudinal velocity (Eq. 38), the mean squared velocity can be derived

41 $$\begin{aligned} {u^\prime }^2=\overline{u^2}=\frac{19\pi }{8}u^2_0 \end{aligned}$$

where $u^\prime$ is the conventional longitudinal turbulence intensity and the sum of natural numbers $\sum \limits _{k=1}^Kk=\frac{K(K+1)}{2}$ is used. Similarly, we have the mean squared lateral velocity,

42 $$\begin{aligned} {v^\prime }^2=\overline{v^2} =\frac{19\pi }{8}v^2_0 \end{aligned}$$

where $v^\prime$ is the conventional lateral turbulence intensity. The mean squared turbulent velocities are constant proportional to the perturbation velocity squared, which is consistent to experimental observations and the general convention in turbulent flows^4–7^.

Taylor correlation functions have been referred to by different names, such as the Taylor one-dimensional energy spectrum, Taylor hypothesis or the two-point second-order moment. Nevertheless, Taylor correlation functions represent turbulent flows with the most succinct form and are reproduced consistently in many experiments of turbulent flows. In fact, the simple relation of the correlations with turbulent velocities attracted the attention of researchers such as^5,19,20^ and many others. However, no such velocity is available so far in public domain, and known to the author. Here, the emphasis is the physics that leads to velocities Eqs. (38) and (39) and the subsequent correlation functions. Acoustics and turbulent flow are two common phenomena and share the same fundamental physics at various stages. When a motion takes place isentropically, superfluid, sound and turbulence will occur. Without the introduction of this physics, it will be difficult to circumvent the longitudinal and lateral velocity distributions.

By definition, Taylor’s correlations are denoted by^2^

43 $$\begin{aligned} \begin{aligned} R_x\left( \frac{x}{U}\right)&= \frac{\overline{u(t)u(t+\frac{x}{U})}}{\overline{u^2}} \\&=\frac{4\pi ^2 u_0^2}{K\overline{u^2}}\lim _{T \rightarrow \infty }\sum \limits _{k=1}^K\int ^T_0\left( 3k\cos {2\pi kt} \right. \\& \quad \left. -4k\sin {2\pi kt}\right) e^{-2\pi kt} \\& \quad \left[ 3k\cos {2\pi k(t+\frac{x}{U})} \right. \\& \quad \left. -4k\sin {2\pi k(t+\frac{x}{U})}\right] e^{-2\pi k(t+\frac{x}{U})}dt \end{aligned} \end{aligned}$$

44 $$\begin{aligned} \begin{aligned} R_y\left( \frac{x}{U}\right)&= \frac{\overline{v(t)v(t+\frac{x}{U})}}{\overline{v^2}} \\ {}&=\frac{4\pi ^2 v_0^2}{L\overline{v^2}}\lim _{T \rightarrow \infty }\sum \limits _{l=1}^L\int ^T_0\left( 3l\cos {2\pi lt} \right. \\& \quad \left. -4l\sin {2\pi lt}\right) e^{-2\pi lt} \\& \quad \left[ 3l\cos {2\pi l(t+\frac{x}{U})} \right. \\& \quad \left. -4l\sin {2\pi l(t+\frac{x}{U})}\right] e^{-2\pi l(t+\frac{x}{U})}dt \end{aligned} \end{aligned}$$

where *U* is the longitudinal velocity of the mean flow and *x* is the distance between two points where turbulent velocity is measured and $\frac{x}{U}$ has the unit of time, and $\frac{x}{U}$ can be replaced by any variable equivalent to time, for example, time delay $\tau$^21^ or any nondimensional variable of time such as $\frac{U\tau }{M}$^22^. The integration of Eq. (43), similar to obtaining the mean square velocity in Eq. (41), yields the following expression:

45 $$\begin{aligned} \begin{aligned} R_x\left( \frac{x}{U}\right)&= \frac{2}{19}\sum \limits _{k=1}^K\left( 19\cos {2\pi k\frac{x}{U}} \right. \\& \quad \left. -23\sin {2\pi k\frac{x}{U}}\right) k e^{-2\pi k\frac{x}{U}} \end{aligned} \end{aligned}$$

Similarly, the lateral correlation function can be obtained from Eq. (44) as follows

46 $$\begin{aligned} \begin{aligned} R_y\left( \frac{x}{U}\right)&= \frac{2}{19}\sum \limits _{l=1}^L\left( 19\cos {2\pi l\frac{x}{U}} \right. \\& \quad \left. -23\sin {2\pi l\frac{x}{U}}\right) le^{-2\pi l\frac{x}{U}} \end{aligned} \end{aligned}$$

where both correlation functions are identical in form. The difference between the longitudinal and lateral correlations lies in the fact that since the axial flow dominates, the amplitude of longitudinal velocity is much higher than that of lateral velocity; as the result, the decay of the lateral velocity is much faster. According to (9), the wave composition of the lateral velocity is influenced by the summation of temporal and axial mode shapes and is likely different from either one of them.

Briefly, by utilizing velocity distributions, we analytically derived the correlation functions and basic statistical properties observed experimentally. In the next section, we determine the amplitude and frequency factors of these correlations and validate them with experimental data.

## Validation

In this section, the validation is organized into three parts. First, we demonstrate that the formulas can be curve fitted to the desired precision with two selected experimental datasets, which shows that the theory and formulas phenomenologically describe a turbulent flow. Second, we compare the longitudinal correlation with other known experimental datasets. The comparison shows that the formulas capture the key features of those experiments and that there are appreciable variations between experiments. Third, we apply the longitudinal correlation to a spatial evolution dataset and show that the theory describes the correlation in the ranges of both positive and negative eigenvalues in space. Although the amplitude and frequency factors are curve-fitted in the range of positive eigenvalues, the comparison shows good agreement in the negative eigenvalue region, especially near the peak. The good agreement between the experimental data and theory solidifies the confidence in the theory. In the next section, we use the turbulent velocities and correlations to gain insights into the physics of turbulence. As complex as turbulence, one must recognize that no validation can possibly be accomplished by one manuscript. With that in mind, we concede that the validation here is just the beginning of the process and is limited.

It is known that a turbulent flow is a formidable problem in complexity and difficulty. There will be little argument on the fact that we have to solve it in multiple steps. As well, it is almost sure that as we understand more, the convoluted process to obtain Eqs. (45) and (46) will be simplified but for now, it has taken a big portion of the manuscript. As soon as we had access to Eqs. (45) and (46) and the experimental data from^2,21,23–25^, it was the natural reaction for us to curve-fit this universal correlation function. We have to remember that Eqs. (45) and (46) are systematically derived, whose realization by the curve fitting is based on their universality. During the fitting process, we have also learnt significantly the properties of them. Especially, there are many combinations of coefficients that can produce the same correlation functions, which is the fundamental reason that the Taylor correlation functions are universal. In order to solve these coefficients, we immediately started to work on the mode shapes and spatial distribution, which, we believe, will lead the determination of these coefficients analytically. Soon, we found that the work on the spatial eigenfunctions is so extensive that it should be kept in a separate manuscript. More importantly, we discovered that the solutions from the negative eigenvalues produce the correlation functions on the negative axis, which is equivalent to validate the correlation functions on the negative axis with a blind comparison. Such a comparison has much higher fidelity than a curve-fitting.

Considerations and findings above make us decide to check in with the general community and experts in turbulence and turbulent flow and appreciate the feedbacks and directions that we will be offered during and after the publishing process.

Beyond the considerations above, we believe that there are several benefits for the general scientific and engineering community if we check in right now. A function form of the correlation functions may be valuable for some disciplines so that a reader can apply them to approach the theoretical and technical challenges that one is facing. A scientist, engineer or technician can use the conclusions on the negative eigenvalues to check the instrumentation or software that conducts such computation and correct them if necessary. It promotes the experiments or publishing data on a much wider range of the time delay so that we can learn more about turbulence from the theory and experiments since the current datasets are limited to small time delays.

With the property of scalable eigenvalues in Eqs. (9), we assume that the turbulent velocities can be multiplied by two constants on real and imaginary parts, and they are the amplitude and frequency factors,

47 $$\begin{aligned} \begin{aligned} u&= 2\pi u_0\sum \limits _{k=1}^K \alpha _k{k}\left( 3\cos {2\pi {\beta _k kt}} \right. \\&\quad \left. -4\sin {2\pi {\beta _k kt}}\right) e^{-2\pi {\alpha _k kt}} \end{aligned} \end{aligned}$$

Similarly, we can derive the lateral turbulent velocity,

48 $$\begin{aligned} \begin{aligned} v&= 2\pi v_0\sum \limits _{l=1}^L\alpha _l l\left( 3\cos {2\pi {\beta _l l t}} \right. \\&\quad \left. -4\sin {2\pi {\beta _l lt}}\right) e^{-2\pi {\beta _l lt}} \end{aligned} \end{aligned}$$

where $\alpha _k$ and $\alpha _l$ are amplitude factors and $\beta _k$ and $\beta _l$ are frequency factors, which allow the correlation functions to tolerate small variations from experiments and mode shapes. The derivation of the correlation functions with these factors is identical to obtaining Eqs. (45) and (46), which are given as follows:

49 $$\begin{aligned} \begin{aligned} R_x\left( \frac{x}{U}\right)&= \varphi \sum \limits _{k=1}^K \left[ (34\alpha ^2_k+25\beta ^2_k - 40\alpha _k\beta _k)\cos {2\pi \beta _{k}k\frac{x}{U}} \right. \\&\quad \left. -(36\alpha ^2_k+24\beta ^2_k- 37\alpha _k\beta _k)\sin {2\pi \beta _{k}k\frac{x}{U}}\right] \\&\quad \frac{\alpha _k k}{\alpha ^2_k+\beta ^2_k}e^{-2\pi \alpha _k k\frac{x}{U}} \end{aligned} \end{aligned}$$

50 $$\begin{aligned} \begin{aligned} R_y\left( \frac{x}{U}\right)&= \psi \sum \limits _{l=1}^L \left[ (34\alpha ^2_l+25\beta ^2_l -40 \alpha _l\beta _l)\cos {2\pi \beta _{l}l\frac{x}{U}}\right. \\&\quad \left. -(36\alpha _l^2 +24\beta _l^2-37 \alpha _l\beta _l)\sin {2\pi \beta _{l}l\frac{x}{U}}\right] \\&\quad \frac{\alpha _l l}{(\alpha ^2_l+\beta ^2_l)}e^{-2\pi \alpha _l l\frac{x}{U}} \end{aligned} \end{aligned}$$

where $\varphi$ and $\psi$ are from $R_x(0)$ and $R_y(0)$ to normalize $R_x$ and $R_y$,

$$\begin{aligned} \varphi= {} \frac{1}{\sum \limits _{k=1}^K \frac{\alpha _k }{\alpha ^2_k+\beta ^2_k}(34\alpha ^2_k+25\beta ^2_k - 40\alpha _k\beta _k)}\\ \psi= {} \frac{1}{\sum \limits _{l=1}^L \frac{\alpha _l}{(\alpha ^2_l+\beta ^2_l)}(34\alpha ^2_l+25\beta ^2_l -40 \alpha _l\beta _l)} \end{aligned}$$

and $\alpha _1$ and$\beta _1$ are amplitude factors and frequency factors at $k=1$ and $l=1$, respectively, which are the same as these given in Eqs. (47) and (48). When $\alpha _k=1$ and $\beta _k=1$, Eq. (49) is reduced to Eq. (45) as expected.

We compare Eqs. (49) and (50) with several known experimental datasets in the public domain^2,21–25^. In general, calculations from Eqs. (49) and (50) agree very well with data. As pointed out by^21^, correlations from each dataset are slightly different from each other despite having the same appearances and trends due to the phase angles of data collected. The small disparity is due to the dependence of the eigenfunctions on spatial mode shapes through Eq. (9). Hence, we allow the frequency and amplitude factors to float slightly and report them along the comparisons presented here.

**Figure 2:**
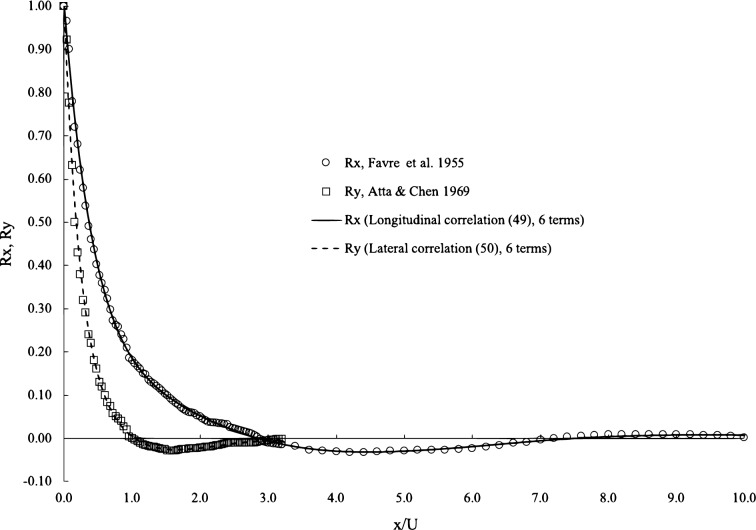
Curve-fitting of longitudinal and lateral correlations. Experimental data from^22^ for the longitudinal correlation andfrom^25^ for the lateral correlation.

The longitudinal correlation is curve-fitted to the experimental data by^22^ and the lateral correlation to the experimental data by^25^. They are both wind tunnel after-grid turbulence. The longitudinal correlation data from^22^ have a very wide range of x/U from 0 to more than 100, and the curve-fitting here uses the data from 0 to 70 due to the quality of data beyond 70. To avoid choosing the experimental data from the same source and the preference for the latest dataset, the later experimental data by^25^ are selected for the curve-fitting for the lateral correlations. For each correlation, we use two series with 6 terms or terms to curve fit. The error is calculated by the sum of the differences between experimental data and theory, the desired and calculated errors of all curve fits are less than 1 $\%$, and there is no visual difference between 6 terms and 10 terms, as illustrated in Fig. 2. The amplitude and frequency factors from curve fit are given in Table 1 for these 6 terms and Table 2 for these 10 terms. It is interesting to observe that although the errors are very low, these factors from 6 terms are different from those in 10 terms. When the range of the data is larger, the differences between these factors are larger, for example, in the longitudinal correlation. Although it has a small range in the lateral data, there are some appreciable differences between the factors in 6 terms and 10 terms. The variation in these factors is observed in the trials of curve fitting. With the same level of errors, these factors can change slightly here and there without changing the shape of the curves. This suggests that there are infinite combinations of these factors to yield the same or slightly different correlation functions. In other words, the functions and forms of the correlations Eqs. (49) and (50) will not change regardless of variations in these factors. In physics, such observation is also consistent with the observation that the Taylor correlation functions vary slightly from one test to another. Additionally, it was explained previously that these factors are functions of the mode shape and the spatial distribution of nodes and antinodes. A vivid presentation of this kind of phenomena is the Chladni pattern, where at the same resonance frequency, there are many different patterns due to many radial and angular modes in a solid plate.

**Table 1:** Amplitude and frequency factors by curve-fitting 6 term correlations.

k, l	Longitudinal	Factors	Lateral	Factors
	$\alpha _k$	$\beta _k$	$\alpha _l$	$\beta _l$
1	1.2569E−1	2.6979E−3	7.8901E−1	3.3731E−3
2	4.5120E−5	1.9547E−1	7.0859E−3	1.5318E−1
3	7.8979E−3	7.9215E−3	5.2742E−2	4.0467E−2
4	6.5119E−3	2.3032E−2	1.1903E−1	0.000E+0
5	8.7398E−3	1.5176E−2	1.2666E−2	2.7643E−2
6	5.3438E−2	2.4766E−2	1.8769E−2	2.8635E−2

**Table 2:** Amplitude and frequency factors by curve-fitting 10 term correlations.

k, l	Longitudinal	Factors	Lateral	Factors
	$\alpha _k$	$\beta _k$	$\alpha _l$	$\beta _l$
1	3.4288E−1	2.6621E−3	7.0778E−1	0.000E+0
2	7.7780E−5	2.0097E−1	2.0471E−3	7.9155E−1
3	4.7935E−3	2.9699E−2	5.4469E−2	1.3868E−2
4	8.4886E−3	2.8787E−2	1.8617E−1	1.7235E−4
5	5.4795E−3	1.3648E−2	1.2544E−2	2.5580E−2
6	1.9209E−2	3.3624E−2	1.9570E−2	2.9885E−2
7	1.2720E−2	2.5283E−3	1.9888E−2	2.5985E−2
8	2.0450E−3	4.4791E−3	2.0271E−2	2.1731E−2
9	1.2401E−2	5.9460E−4	2.0877E−2	1.1414E−2
10	2.7720E−4	1.0524E−3	3.5836E−2	1.4326E−2

**Figure 3:**
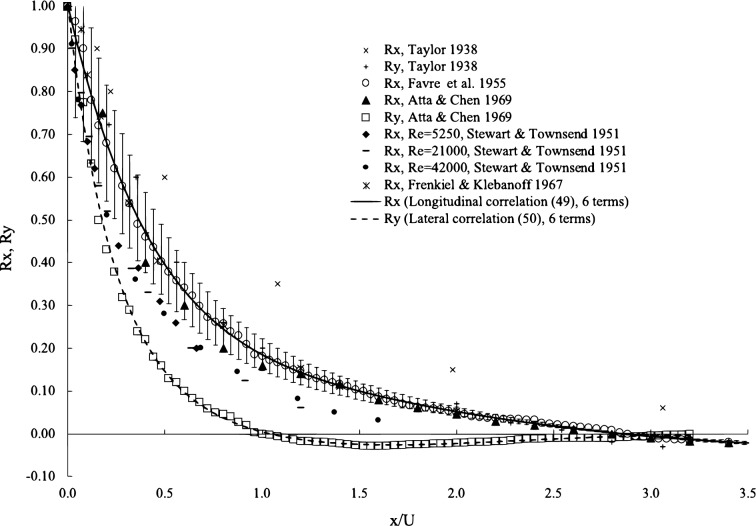
Comparisons of longitudinal and lateral correlations with experimental data. Experimental data from Refs. ^2,23^ for Taylor, Ref. ^22^ for Favre et al., Ref. ^25^ for Atta and Chen, Ref. ^21^ for Stewart and Townsend, and Ref. ^24^ for Frenkiel and Klebanoff.

In Fig. 3, we compare the 6-term correlation functions with several experimental datasets in public domain. We included the dataset where the original correlation functions were discovered. Since the physics and derivations indicate that both lateral and longitudinal correlations bear the same fundamentals, in Fig. 3, we included the comparison of the longitudinal correlation with the experimental data only. On the correlation curve, we add 20$\%$ error bars to visualize the spread, and the scattering of data has the same trend as the error bars. Higher correlation coefficients also have larger errors. The correlations by Taylor’s data^2,23^ are consistently higher and those by Stewart and Townsend are consistently lower^21^, while those from Frenkiel and Klebanoff^24^ are in good agreement with the theory and Favre et al.^22^ and Atta and Chen’s experimental data^25^. The longitudinal correlation crosses the abscissa at some points and becomes negative and then returns to positive again. When the longitudinal correlation reaches the first zero at approximately 2.9 and the lateral correlation at approximately 1.0, the experimental datasets are coherent themselves, and the theory agrees with these datasets very well. Taylor and Simmons’ data do not cross the abscissa, while Stewart and Townsend’s data cross at lower values. Since the delay parameter is defined with certain freedom, it is unclear whether those having larger variations are due to the choices of correlation parameter or other uncertainties.

**Figure 4:**
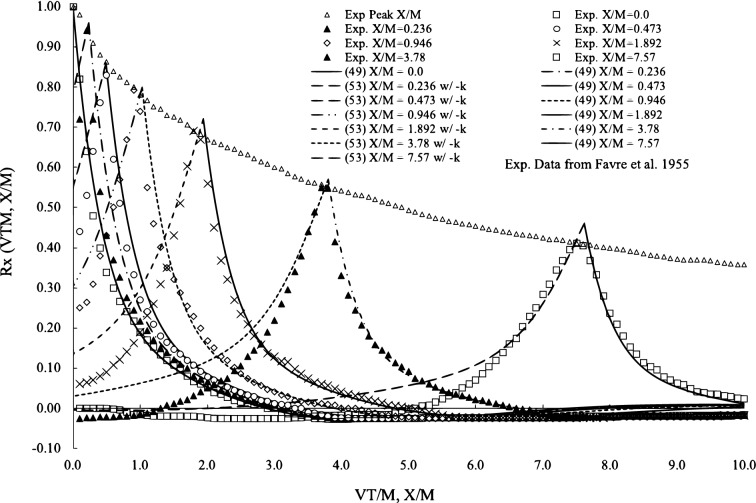
Comparisons of longitudinal correlation and experimental data with both time and space delays. Experimental data is from^22^. $\frac{VT}{M}$ refers to time delay, and $\frac{x}{M}$ refers to space delay. Each peak is measured at a specific $\frac{x}{M}$ and the correlation is plotted with curves on both sides as positive and negative time delay by $\frac{VT}{M}$.

In Eq. (9), it is possible that the eigenvalue of the temporal component is negative, which is also a legitimate solution of turbulent velocities. In that case, the velocities of negative eigenvalues have the forms of

51 $$\begin{aligned} u^{-}= 2\pi u_0\sum \limits _{k=-1}^{-K}{\alpha _k{k}\left( 3\cos {2\pi {\beta _k kt}}-4\sin {2\pi {\beta _k kt}}\right) e^{2\pi {\alpha _k kt}}} \end{aligned}$$

52 $$\begin{aligned} v^{-}= 2\pi v_0\sum \limits _{l=1-}^{-L}\alpha _l l\left( {3\cos {2\pi {\beta _l l t}}-4\sin {2\pi {\beta _l lt}}}\right) e^{2\pi {\beta _l lt}} \end{aligned}$$

where $u^{-}$ and $v^{-}$ are velocities with negative eigenvalues of Eqs. (47) and (48). When we correlate Eq. (47) with Eqs. (51) or (48) with Eq. (51), we find the correlation functions in the negative parameter as follows:

53 $$\begin{aligned} \begin{aligned} R_{-x}\left( \frac{x}{U}\right)&= \varphi \sum \limits _{k=1}^K \left[ (34\alpha ^2_k+25\beta ^2_k - 40\alpha _k\beta _k)\cos {2\pi \beta _{k}k\frac{x}{U}} \right. \\&\quad \left. -(36\alpha ^2_k+24\beta ^2_k- 37\alpha _k\beta _k)\sin {2\pi \beta _{k}k\frac{x}{U}}\right] \\&\quad \frac{\alpha _k k}{\alpha ^2_k+\beta ^2_k}e^{2\pi \alpha _k k\frac{x}{U}}\\&\quad for \quad \frac{x}{U}<0 \end{aligned} \end{aligned}$$

54 $$\begin{aligned} \begin{aligned} R_{-y}\left( \frac{x}{U}\right)&= \psi \sum \limits _{l=1}^L \left[ (34\alpha ^2_l+25\beta ^2_l -40 \alpha _l\beta _l)\cos {2\pi \beta _{l}l\frac{x}{U}}\right. \\&\quad \left. -(36\alpha _l^2 +24\beta _l^2-37 \alpha _l\beta _l)\sin {2\pi \beta _{l}l\frac{x}{U}}\right] \\&\quad \frac{\alpha _l l}{(\alpha ^2_l+\beta ^2_l)}e^{2\pi \alpha _l l\frac{x}{U}} \\&\quad for \quad \frac{x}{U}<0 \end{aligned} \end{aligned}$$

where for convenience a transformation of $k=-k$ and $l=-l$ has made for Eqs. (53) and (54) and $\varphi$ and $\psi$ are the same in Eqs. (49) and (50). The correlations Eqs. (53) and (54) only exist in the negative axis, $\frac{x}{M}<0$. In the dataset, the corresponding factors in Eqs. (49) and (50) should hold. In Fig. 4, we compare the results from Eqs. (49) and (53) to the experimental dataset by^22^. The experiments were supposed to demonstrate the time and space delay simultaneously. The time parameter $\frac{VT}{M}$ has velocity *V*, time *T*, and mesh size *M* for turbulence generation, and $\frac{x}{M}$ is the space parameter. Since they are dimensionless, the space and time delay can be plotted on the same scale. Each peak represents a time delay at a given space X/M, except for the top dataset. At each peak, the curve on the right is calculated from the correlation of Eq. (49), and the curve on the left is from Eq. (53) by opposing correlation, where asymmetricity is visible. For all peaks, the correlations on both sides from the theory agree very well with the experimental data. At turning regions, the negative-side correlation does not have as sharp an elbow as the data, although it meets the data again further away. Since in the curve fitting, we purposely blind the knowledge of the negative-side correlation, the good agreement in Fig. 4 is very encouraging.

In summary, we compared calculations from Eqs. (49), (50) and (53) to experimental data in isotropic turbulent flows. We obtained two sets, 6 terms and 10 terms, of amplitude and frequency factors by curve fitting to two different data sources. These factors are tabulated in Tables 1 and 2, and the curve fitting of all correlations is less than 1$\%$ error. We then compare the correlations to other datasets. When the first zeros of the experimental data are consistent, the agreements are very good, and a large deviation is observed when the first zeros are off. We also evaluated the evolution of the turbulent distribution in space and compared the calculation to the experimental dataset. The correlation between velocities with the opposite eigenvalues makes up the negative axis and is axisymmetric to the correlation on the positive axis. The correlations from both positive and negative parameters agree very well with the experimental data in space and time delay. We are very encouraged by the results, and the validation work will continue beyond this manuscript.

For convenience, we tabulate various velocities, their physical meaning and the equation numbers where they appeared in Table 3.

**Table 3:** Velocity, physical meaning and equations appeared.

No.	Symbol	Physical meaning	Equations
1	$\textbf{u}$	Velocity vector of superfluid	(1)
2	*u*, *v*	Velocities of resonant superfluid	(4), (5)
3	$u_h$	Homogeneous solution of resonant superfluid	(6)
4	$u_p$	Nonhomogeneous solution of resonant superfluid	(11)
5	$u_s$, $v_s$	Steady-state velocity of resonant superfluid	(15)–(17)
6	$u^\dagger$, $v^\dagger$	Perturbation velocity of regular fluid	(24), (25)
7	$u^\dagger _0$, $v^\dagger _0$	Amplitudes of perturbation	(24), (25)
8	$\pitchfork _1$, $\pitchfork _2$	Dirac comb in positive and negative directions, positive and negative impulses	(30),(31)
9	$\bar{u}$	Arithmetic mean velocity	(40)
10	${u_\prime }^2$, $\bar{u^2}$	Longitudinal mean squared velocity of turbulent flows	(41)
11	${v_\prime }^2$, $\bar{v^2}$	Lateral mean squared velocity of turbulent flows	(42)
12	U	Longitudinal velocity of the mean flow	(43)
13	$u^{-}$, $v^{-}$	Longitudinal and lateral velocities of resonant superfluid with negative eigenvalues	(51), (52)

## Discussion

In this section, we discuss the essential elements and the results developed through the manuscript. Our discussions focus on three aspects that, we believe, are significant. They are the fundamental physics, correlations and turbulent velocities. The hypothesis that turbulence is the transient part of superfluid at resonance is a fair new concept, where further discussion will help to clarify more. Since we have an analytic representation of the Taylor correlation functions, we can explore the certain specifics in the correlations that were inaccessible. Through validation, the turbulent velocities are also determined, which provides an opportunity to learn the properties of turbulent velocities.

The fundamental premise of the theory is that in a body of fluid, the physics of fluid motion is different from that in a viscous fluid when it does not momentarily change the entropy. The excess entropy is defined to quantify the temporal and spatial conditions in Eq. (20)

20 $$\begin{aligned} \delta {s}=s_{ND}-s_D \end{aligned}$$

where $s_{D}$ and $s_{ND}$ are the mass density of entropy of a dissipative fluid and a non-dissipative fluid, respectively. In this framework, a regular or viscous fluid turns into a superfluid when excess entropy is zero. A superfluid is a fluid at a special thermodynamic state, the isentropic state. The excess entropy can be calculated by the differential equation derived from energy conservation:

21 $$\begin{aligned} \frac{\partial \delta {s}}{\partial t}+\textbf{u}^\dagger \cdot \nabla \delta {s} =\frac{1}{\rho T}{\mathbf {\nabla }}\cdot \textbf{q}-\frac{\varepsilon }{\rho T} \end{aligned}$$

If there is no conduction and the dissipation rate $\epsilon$ becomes zero, at that moment or instance, the fluid is locally superfluid. Because of the second law of thermodynamics, at that moment, the motion is isentropic, which has no viscous dissipation or thermal conduction. Therefore, at that moment, the governing equations are the Euler equations, not the Navier–Stokes equations. In an oscillatory motion, a parcel of fluid is repetitively in the isentropic state, which drives the fluid into resonance, which describes the differential equations for superfluid at resonance:

47 $$\begin{aligned} u = 2\pi u_0\sum \limits _{k=1}^K{\alpha _k{k}\left( 3\cos {2\pi {\beta _k kt}}-4\sin {2\pi {\beta _k kt}}\right) e^{-2\pi {\alpha _k kt}}} \end{aligned}$$

48 $$\begin{aligned} v = 2\pi v_0\sum \limits _{l=1}^L\alpha _l l\left( {3\cos {2\pi {\beta _l l t}}-4\sin {2\pi {\beta _l lt}}}\right) e^{-2\pi {\beta _l lt}} \end{aligned}$$

and for negative eigenvalues

51 $$\begin{aligned} u^{-} = 2\pi u_0\sum \limits _{k=-1}^{-K}{\alpha _k{k}\left( 3\cos {2\pi {\beta _k kt}}-4\sin {2\pi {\beta _k kt}}\right) e^{2\pi {\alpha _k kt}}} \end{aligned}$$

52 $$\begin{aligned} v^{-} = 2\pi v_0\sum \limits _{l=1-}^{-L}\alpha _l l\left( {3\cos {2\pi {\beta _l l t}}-4\sin {2\pi {\beta _l lt}}}\right) e^{2\pi {\beta _l lt}} \end{aligned}$$

**Figure 5:**
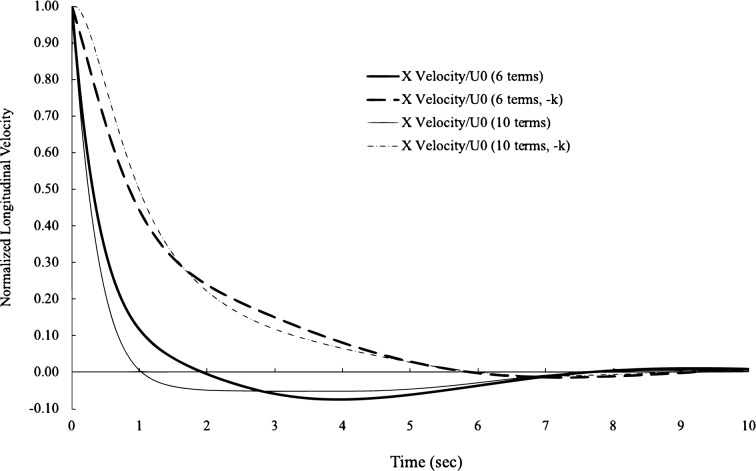
Normalized longitudinal turbulent velocity with positive and negative eigenvalues with 6 and 10 terms.

**Figure 6:**
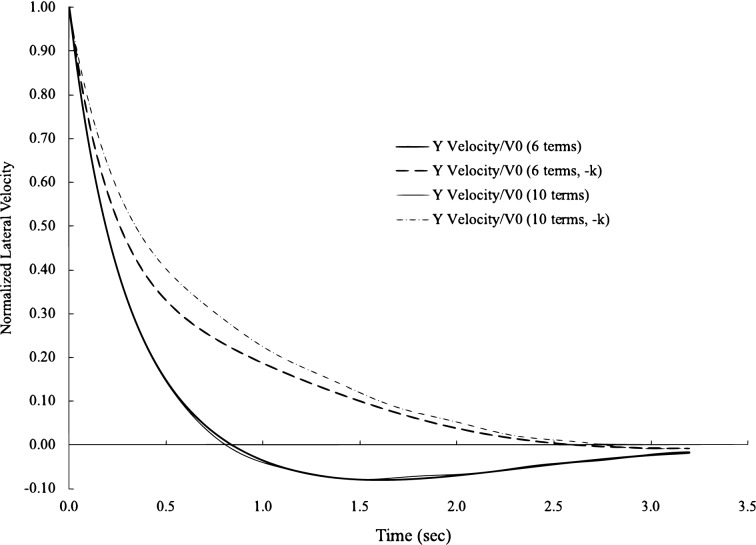
Normalized lateral turbulent velocity with positive and negative eigenvalues with 6 and 10 terms.

It is necessary to note that the velocities with negative eigenvalues are not negative velocities and can be in the same direction as those with positive eigenvalues. The asymmetric nature of correlations between positive–positive eigenvalues and positive–negative eigenvalues shows us that a complete set of velocity solutions should include both positive and negative eigenvalues, which are usually ignored in practice. In Figs. 5 and 6, the significance of negative eigenvalues becomes evident. In Fig. 5, we plot the longitudinal velocity with the amplitude and frequency factors in 6 terms and 10 terms. Along with the positive eigenvalues, we also plot the longitudinal velocity with the negative eigenvalues. Differences between the heavy and light solid lines are from the different numbers of terms for the longitudinal velocity with positive eigenvalues. Differences between the heavy and light broken lines are for the negative eigenvalues. Although there is no visual difference in correlation functions with 6 or 10 terms, the differences are larger in velocities between 6 or 10 terms, which indicates that the correlations are less sensitive to the variations in velocity. However, the differences between positive and negative eigenvalues are more pronounced than those from the number of terms. The disparity between the velocities with positive or negative eigenvalues shows the significance of velocity with negative eigenvalues. In Fig. 6, we plot similar information for the lateral velocity, and the number of terms makes little difference in the velocities with either positive or negative eigenvalues. However, the difference due to positive or negative eigenvalues is substantial.

In Fig. 5, the longitudinal velocity is at its maximum at $t=0$ due to the impulse from regular fluid. After approximately half sec, the negative impulse acts on the same parcel fluid and accelerates the velocity decay. After approximately 2 s, the longitudinal velocity becomes zero momentarily (by the heavy solid line) and turns negative, which means that the longitudinal turbulent velocity moves in the opposite direction. The velocity turns positive again between 7 and 8 s. The physical mean of the correlation function is the summation of the magnitude in one profile in Fig. 5 multiplied by the magnitude in another profile at a given distance of x/U. When the second velocity profile moves into the negative velocity range of the first velocity profile, the summation can become zero or negative, which results in a negative region at approximately 2.9 in Fig. 2 for the longitudinal correlation. In that negative region, the velocity of one fluid parcel is moving in the opposite direction of the other. The lateral velocity has a much lower amplitude; hence, the occurrence of the zero point is much earlier (Fig. 6), where the lateral velocity reaches zero at approximately 0.76 s while the longitudinal velocity is at approximately 2 s.

**Figure 7:**
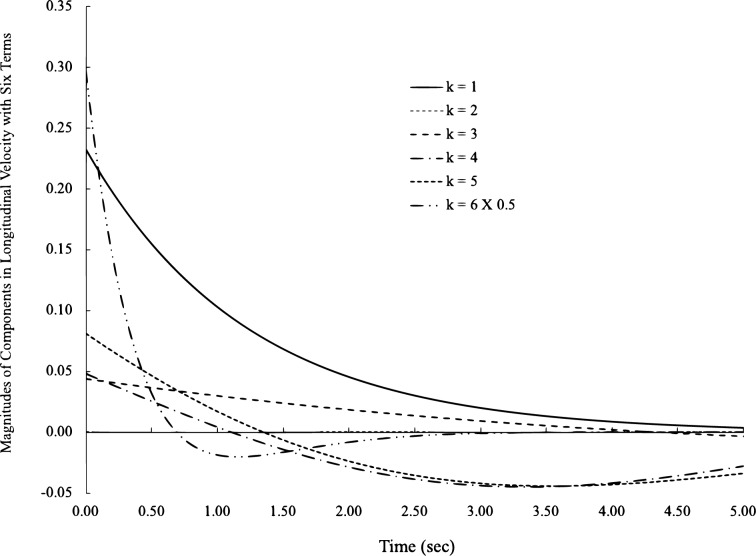
Contributions of each term to longitudinal velocity with 6 terms.

When we plot them, the contribution from each term of the longitudinal velocity can be analyzed individually in Fig. 7. In a 6-term longitudinal velocity, the term from $k=6$ has the largest variation, and the term of $k=2$ has the smallest contribution due to its extremely low amplitude factor ($4.5E-5$). At approximately $t=2$ s, terms 1 and 3 are almost equivalent to the sum of terms 4, 5 and 6, where zero velocity is observed. Although these factors change with mode shapes and frequencies, which will change the composition of these terms, the structure and characteristics of turbulent velocities and correlations made of elemental functions will not change.

## Conclusions

In this manuscript, we analytically derived, fitted and validated Taylor correlation functions by assuming turbulence as a phenomenon of superfluids at resonance.

A recent study showed that the motion of a regular fluid can experience a different thermodynamic state and be excited locally when an oscillation persists. At these excitation sites, the change in the governing equations in velocity leads to the theory of turbulence formation in this manuscript. From the differential equations of superfluids at resonance, we derived the distribution of longitudinal and lateral velocities in a three-dimensional isotropic turbulent flow.

By assuming a small sinusoidal perturbation in the longitudinal direction on the regular fluid side, we obtained the Dirac combs of velocity impulses on the boundary of the second law. Through Fourier integration and coefficients, these Dirac combs close the boundary conditions in longitudinal and lateral turbulent velocities. These velocity profiles produce mean velocity and root mean square velocities that are consistent with those from statistics and experimental data. Taylor correlation functions for longitudinal and lateral velocities are subsequently derived. The correlation functions are represented by series, and each term in the series consists of two elementary functions: trigonometric and exponential functions. The trigonometric functions are attributed to small negative regions repeated along the temporal or spatial distances observed experimentally. The exponential function dominates the behavior of correlation functions and results in descending power laws, reported in various forms. The fundamental reason that any turbulence fits Taylor’s 1D correlation lies in the fact that they are in resonance, and therefore, all spatial and temporal variables can be expressed linearly in an eigenfunction. Here, we want to emphasize that there is no compromise on the nonlinearity of Reynolds stresses since the linearity of the governing equations of resonant superfluid does not change the nature of Reynolds stresses at all.

Through validation, it is found that the solutions of negative eigenvalues are different from those of positive eigenvalues. Experimental data and theory suggest that a complete solution shall include these from the negative eigenvalues.

In Eqs. (47), (48), (49), (50), (51), (52), (53), and (54), turbulent velocities and correlation functions are presented in fairly simple form as a function of time only, although they relate to complicated mode shapes in space. This complexity is observed through the fact that Taylor correlation functions vary somewhat from one test to another. The nodes, antinodes and amplitudes of excitation are dependent on geometry, fluid properties and boundary conditions. However, once we cast them into the function of time as we have done in this manuscript, the general formulas of turbulent velocities and correlation functions are analytically determined.

As soon as we adopt the concept of the boundary of the second law, a completely new physics dealing with non-dissipative processes unfolds, which covers nearly all subjects of science and provides consistent solutions for many outstanding problems. The examples of solving the steady-state problems in non-dissipative dynamics are given in the previous work on interface thermal resistance and spontaneous thermodynamic cycles. The work presented in this manuscript exemplifies the mathematics on the boundary conditions and solutions of turbulence, disorder, transient dynamics and their correlation functions, in addition to the practical applications of those correlation functions.

## Supplementary Information

Supplementary Information.

## Data Availability

All data relevant to the study are included in the article or uploaded as supplementary information. In addition, the datasets used and/or analyzed during the current study are available from the corresponding author on reasonable request.
