# Supplementary material for: On taylor correlation functions in isotropic turbulent flows
Source: Sci Rep. 2023 Mar 8;13:3859. doi: 10.1038/s41598-023-30825-3 (PMC9995477; doi:10.1038/s41598-023-30825-3)
Supplement: Supplementary file 1 — Supplementary Information. [file 41598_2023_30825_MOESM1_ESM.pdf]

Supplement I

This Supplement includes closely-related two subsections on the resonance of superfluid. The first is the derivation of the governing equations of superfluid at resonance and the second is the speed of the second sound.

I.1 Resonance of Superfluid

In this Supplement, we derive the governing equations of the resonant superfluid (2) and (3) from the governing equations of (12), (13) and (16) in [1]. The derivation serves as a transition from our previous work on the super heat transfer to the Taylor correlation function in this manuscript. The reasons that we need this transition are two folds. First, although we implemented the technique by Landau on the derivation of the resonant state, we do not use the two-fluid model that Landau and Tisza developed as we have stressed in [1]. A reader may compare the derivation in this Supplement with Landau's to appreciate the differences between this method and Landau's.

The governing equations of superfluid are (12), (13) and (16) in [1] are given below

$$\frac{D\rho}{Dt} + \rho\,\nabla\cdot\mathbf{u} = 0 \qquad \text{(S.1)}$$

$$\rho\frac{D\mathbf{u}}{Dt} = -\nabla p \qquad \text{(S.2)}$$

$$\frac{\partial T}{\partial t} + \mathbf{u}\cdot\nabla\mathrm{T} = -\frac{T}{\rho c_v}\left(\frac{\partial p}{\partial T}\right)_v \nabla\cdot\mathbf{u} \qquad \text{(S.3)}$$

Thermodynamics relation of density, pressure and temperature is given by

$$dp = \left(\frac{\partial p}{\partial \rho}\right)_s d\rho + \left(\frac{\partial p}{\partial s}\right)_\rho ds \qquad \text{(S.4)}$$

and

$$dT = \left(\frac{\partial T}{\partial \rho}\right)_s d\rho + \left(\frac{\partial T}{\partial s}\right)_\rho ds \qquad \text{(S.5)}$$

Since it is an isentropic motion, superfluid follows

$$dp = \left(\frac{\partial p}{\partial \rho}\right)_s d\rho \qquad \text{(S.6)}$$

and

$$dT = \left(\frac{\partial T}{\partial \rho}\right)_s d\rho \qquad \text{(S.7)}$$

Utilize (S.6) for $\frac{\partial \rho}{\partial t}$, (S.1) becomes

$$\frac{1}{\left(\frac{\partial p}{\partial \rho}\right)_s}\frac{\partial p}{\partial t} + \nabla\cdot(\rho\mathbf{u}) = 0 \qquad \text{(S.8)}$$

Take the derivative of (S.8) with respect to time,

$$\frac{1}{\left(\frac{\partial p}{\partial \rho}\right)_s}\frac{\partial^2 p}{\partial t^2} + \nabla\cdot\left[\rho\frac{\partial(\mathbf{u})}{\partial \mathrm{t}}\right] - \nabla\cdot[\mathbf{u}\nabla\cdot(\rho\mathbf{u})] = 0$$

(A.9)

Take a divergence to (S.2),

$$\nabla\cdot\left[\rho\left(\frac{\partial \boldsymbol{u}}{\partial t}\right)\right] + \nabla\cdot[(\rho\mathbf{u})\cdot\nabla\mathbf{u}] = -\nabla^2 p \qquad \text{(S.10)}$$

Subtract (S.9) from (S.10),

$$\frac{1}{\left(\frac{\partial p}{\partial \rho}\right)_s}\frac{\partial^2 p}{\partial t^2} - \nabla\cdot[\nabla\cdot(\rho\mathbf{u}\mathbf{u})] = \nabla^2 p \qquad \text{(S.11)}$$

Rearrange (S.11),

$$\frac{\partial^2 p}{\partial t^2} - \left(\frac{\partial p}{\partial \rho}\right)_s \nabla^2 p = \Psi \qquad \text{(S.12)}$$

where $\Psi = \left(\frac{\partial p}{\partial \rho}\right)_s \nabla \cdot [\nabla \cdot (\rho \mathbf{uu})]$ is the nonlinear term. (S.12) is the wave equation for the momentum conservation of superfluid. A formula, similar to (S.12), of aeroacoustics was discovered by [2] and the work has been widely recognized in the sound generation and transmission due to turbulence [3].

Take the derivative to (S.3) with respect to $\mathbf{x}$ and t and subtract the former from the latter,

$$\frac{\partial^2 T}{\partial t^2} - \mathbf{u} \cdot \mathbf{u} \nabla^2 T = \Phi \tag{S.13}$$

where $\Phi = -\frac{1}{\rho}\frac{\tilde{D}\boldsymbol{\rho}}{\tilde{D}t}\left(\frac{\partial T}{\partial t} + \mathbf{u} \cdot \boldsymbol{\nabla} T\right) - \frac{\tilde{D}\mathbf{u}}{\tilde{D}t}\boldsymbol{\nabla} T - \frac{\beta}{\rho}\frac{\tilde{D}T}{\tilde{D}t}\nabla \cdot \mathbf{u} + \frac{\beta T}{\rho}\frac{\tilde{D}}{\tilde{D}t}(\nabla \cdot \mathbf{u})$ and $\beta = -\frac{1}{\hat{C}_V}\left(\frac{\partial p}{\partial T}\right)_V$. (S.13) is the wave equation for the energy conservation of superfluid.

From (S.12) and (S.13), we can construct a two-dimensional dynamical system (p, T) that

$$F(p,T) = \frac{\partial^2 p}{\partial t^2} - \left(\frac{\partial p}{\partial \rho}\right)_s \nabla^2 p \tag{S.14}$$

$$G(p,T) = \frac{\partial^2 T}{\partial t^2} - u^2 \nabla^2 T \tag{S.15}$$

At any arbitrary wavelets,

$$p = p_0 \sin(\mathbf{x} + \mathbf{c_1} t) \text{ and } T = T_0 \sin(\mathbf{x} + \mathbf{c_2} t) \tag{S.16}$$

where c_1 and c_2 are the characteristic speeds of the momentum and energy disturbances. When the dynamical system experiences the disturbances of (S.16), (S.14) and (S.15) produce

$$F(p,T) = \frac{\partial^2 p}{\partial t^2} - \left(\frac{\partial p}{\partial \rho}\right)_s \nabla^2 p = -\left[c_1^2 - \left(\frac{\partial p}{\partial \rho}\right)_s\right] p = \Psi \tag{S.17}$$

$$G(p,T) = \frac{\partial^2 T}{\partial t^2} - u^2 \nabla^2 T = -[c_2^2 - u^2] T = \Phi \tag{S.18}$$

We linearize the dynamical system (S.14) and (S.15) by assuming the nonlinear terms, Ψ and Φ, are constant. In a matrix form, (S.17) and (S.18) is expressed by

$$\begin{bmatrix} \frac{\partial F}{\partial p} & \frac{\partial F}{\partial T} \\ \frac{\partial G}{\partial p} & \frac{\partial G}{\partial T} \end{bmatrix} \begin{bmatrix} p \\ T \end{bmatrix} = \begin{bmatrix} \Psi \\ \Phi \end{bmatrix} \tag{S.19}$$

where $J = \begin{bmatrix} \frac{\partial F}{\partial p} & \frac{\partial F}{\partial T} \\ \frac{\partial G}{\partial p} & \frac{\partial G}{\partial T} \end{bmatrix}$ is the Jacobian of this two-dimensional dynamical system. $\begin{bmatrix} p \\ T \end{bmatrix}$ is the linear variable term and $\begin{bmatrix} \Psi \\ \Phi \end{bmatrix}$ is a non-zero constant term. From (S.17) and (S.18), the Jacobian is readily available,

$$J = \begin{bmatrix} \frac{\partial F}{\partial p} & \frac{\partial F}{\partial T} \\ \frac{\partial G}{\partial p} & \frac{\partial G}{\partial T} \end{bmatrix} = -\begin{bmatrix} c_1^2 - \left(\frac{\partial p}{\partial \rho}\right)_s & 0 \\ 0 & c_2^2 - u^2 \end{bmatrix} \tag{S.20}$$

At resonance, the Jacobian reaches zero by

$$\left[c_1^2 - \left(\frac{\partial p}{\partial \rho}\right)_s\right][c_2^2 - u^2] = 0 \tag{S.21}$$

The condition for resonance of superfluid is given by (S.21) and calculated by

$$c_1^2 = \left(\frac{\partial p}{\partial \rho}\right)_s \tag{S.22}$$

and from (S.7),

$$c_2^2 = -\frac{R\rho}{M}\left(\frac{\partial T}{\partial \rho}\right)_s \tag{S.23}$$

where c_1 is the speed of the first sound and c_2 is the speed of the second sound. R is the universal gas constant and M is the molar mass of the fluid. From (S.16), at resonance, the motion of momentum and energy waves is completely determined by the characteristic speed, not the local velocity of the fluid parcel. In other words, the momentum and energy conservation (S.2) and (S.3) are determined by the characteristic speed by

$$\begin{cases} \rho\left(\frac{\partial \boldsymbol{u}}{\partial t} \pm \boldsymbol{c_1} \cdot \nabla \mathbf{u}\right) = -\nabla p \\ \rho\left(\frac{\partial \mathrm{T}}{\partial t} \pm \boldsymbol{c_2} \cdot \nabla \mathrm{T}\right) = -\frac{T}{c_v}\left(\frac{\partial p}{\partial T}\right)_v \nabla \cdot \mathbf{u} \end{cases} \tag{S.24}$$

where $\boldsymbol{c_1} = \mathrm{c}_1(\boldsymbol{i}+\boldsymbol{j}+\boldsymbol{k})$ and $\boldsymbol{c_1} = \mathrm{c}_2(\boldsymbol{i}+\boldsymbol{j}+\boldsymbol{k})$ are acoustic vectors, where the positive and negative signs indicate the waves propagate in both directions. Since it loses all the dissipative properties, superfluid is nearly incompressible, which means $\nabla \cdot \mathbf{u} = 0$. (S.24) is more conveniently written as

$$\begin{cases} \rho\left(\frac{\partial \boldsymbol{u}}{\partial t} \pm \boldsymbol{c_1} \cdot \nabla \mathbf{u}\right) = -\nabla p \\ \rho\left(\frac{\partial \mathrm{T}}{\partial t} \pm \boldsymbol{c_2} \cdot \nabla \mathrm{T}\right) = 0 \end{cases} \tag{S.25}$$

(S.25) can be integrated into the conventional algebra formulas to calculate the impedance and conductance of the first and the second sound.

I.2 Speed of Second Sound

Here, we show that the speed of the second sound can be determined by the thermodynamic variables as that of the first sound.

The speed of the second sound is given in (S.23),

$$c_2^2 = -\frac{R\rho}{M}\left(\frac{\partial T}{\partial \rho}\right)_s \tag{S.26}$$

where R is the universal gas constant, M is the molar mass of the fluid. By assuming $v = v(T, s)$, the total derivative of specific volume v is given

$$dv = \left(\frac{\partial v}{\partial T}\right)_s dT + \left(\frac{\partial v}{\partial s}\right)_T ds \tag{S.27}$$

For an isentropic process, $ds = 0$ and (S.27) becomes

$$dv = \left(\frac{\partial v}{\partial T}\right)_s dT \tag{S.28}$$

Assuming $v = v(T, p)$

$$dv = \left(\frac{\partial v}{\partial p}\right)_T dp + \left(\frac{\partial v}{\partial T}\right)_p dT \tag{S.29}$$

Divide (S.29) by dT

$$\frac{dv}{dT} = \left(\frac{\partial v}{\partial p}\right)_T \frac{dp}{dT} + \left(\frac{\partial v}{\partial T}\right)_p \tag{S.30}$$

For an isentropic process in (S.29) and divide both sides by v,

$$\frac{1}{v}\left(\frac{\partial v}{\partial T}\right)_s = \frac{1}{v}\left(\frac{\partial v}{\partial p}\right)_T \left(\frac{\partial p}{\partial T}\right)_s + \frac{1}{v}\left(\frac{\partial v}{\partial T}\right)_p \tag{S.31}$$

By definition,

$$\frac{1}{v}\left(\frac{\partial v}{\partial P}\right)_T = -\kappa_T \text{ and } \frac{1}{v}\left(\frac{\partial v}{\partial T}\right)_P = \alpha_p \tag{S.32}$$

where κ_T is the isothermal compressibility and α_p is the isochoric thermal expansion coefficient. With (S.32), (S.31) becomes

$$\frac{1}{v}\left(\frac{\partial v}{\partial T}\right)_s = -\kappa_T \left(\frac{\partial p}{\partial T}\right)_s + \alpha_p \tag{S.33}$$

Consider the relation of derivatives with respect to density and specific volume,

$$\frac{1}{v}\left(\frac{\partial v}{\partial T}\right)_s = -\frac{1}{\rho}\left(\frac{\partial \rho}{\partial T}\right)_s = -\frac{1}{\rho\left(\frac{\partial T}{\partial \rho}\right)_s} \tag{S.34}$$

From (S.34), (S.26) is given

$$c_2^2 = -\frac{R\rho}{M}\left(\frac{\partial T}{\partial \rho}\right)_s = \frac{R}{M}\frac{1}{-\kappa_T\left(\frac{\partial p}{\partial T}\right)_s + \alpha_p} = \frac{R_s}{-\kappa_T\left(\frac{\partial p}{\partial T}\right)_s + \alpha_p} \tag{S.35}$$

where $R_s = \frac{R}{M}$ is the specific gas constant. From Maxwell relations, the following dependence exists

$$\left(\frac{\partial p}{\partial T}\right)_s = \left(\frac{\partial s}{\partial T}\right)_p \left[-\left(\frac{\partial p}{\partial s}\right)_T\right] = \frac{c_p}{T}\left(-\frac{1}{\alpha_p V}\right) = -\frac{\rho c_p}{\alpha_p T} \tag{S.36}$$

Bring (S.36) to (S.35), we obtain

$$c_2^2 = -\frac{R\rho}{M}\left(\frac{\partial T}{\partial \rho}\right)_s = \frac{R_s \alpha_p T}{\kappa_T c_p \rho + \alpha_p^2 T} \tag{S.37}$$

In calculation of (S.37), a factor of $\lambda_0 = 10^{-3}$ shall be applied to the first term at the denominator as illustrated in [1],

$$c_2^2 = -\frac{R\rho}{M}\left(\frac{\partial T}{\partial \rho}\right)_s = \frac{R_s \alpha_p T}{\lambda_0 \kappa_T c_p \rho + \alpha_p^2 T} \tag{S.38}$$

where $R_s = \frac{R}{M}$ is the specific gas constant, κ_T is the isothermal compressibility, α_p is the isochoric thermal expansion coefficient, c_p is the isobaric specific heat and $\lambda_0 = 10^{-3}$.

Bibliography

[1] W. Chen, "Heat transfer at speed of sound," *International Journal of Heat and Mass Transfer,* vol. 177, no. 10, pp. 1-13, 2021.

[2] M. Lighthill, "On sound generated aerodynamically I. General theory," *Proceedings of the Royal Society A,* vol. 211, no. 1107, pp. 564-587, 1952.

[3] M. Lighthill, "On Sound generated aerodynamically II. Turbulence as a source of sound," *Proceedings of the Royal Society A,* vol. 222, no. 1148, pp. 1-32, 1954.
